# HLAs associated with perampanel-induced psychiatric adverse effects in a Korean population

**DOI:** 10.1038/s41598-020-70601-1

**Published:** 2020-08-12

**Authors:** Yoonhyuk Jang, Tae-Joon Kim, Jangsup Moon, Tae-Won Yang, Keun Tae Kim, Byeong-Su Park, Jung-Ah Lim, Jin-Sun Jun, Soon-Tae Lee, Keun-Hwa Jung, Kyung-Il Park, Ki-Young Jung, Kon Chu, Sang Kun Lee

**Affiliations:** 1 Laboratory for Neurotherapeutics, Department of Neurology, Comprehensive Epilepsy Center, Biomedical Research Institute, Seoul National University Hospital, 101 Daehak-ro, Jongno-gu, Seoul, 110-744 South Korea; 2 Department of Neurology, Ajou University School of Medicine, Suwon, South Korea; 3 Rare Disease Center, Seoul National University Hospital, Seoul, South Korea; 4 Department of Neurology, Gyeongsang National University Changwon Hospital, Gyeongsang National University School of Medicine, Changwon, South Korea; 5 Department of Neurology, Keimyung University Dongsan Medical Center, Daegu, South Korea; 6 Department of Neurology, Ulsan University Hospital, Ulsan, South Korea; 7 Department of Neurology, Chamjoeun Hospital, Gwangju, South Korea; 8 Department of Neurology, Kangnam Sacred Heart Hospital, Hallym University College of Medicine, Seoul, South Korea; 9 Department of Neurology, Seoul National University Hospital Healthcare System Gangnam Center, Seoul, South Korea

**Keywords:** Haplotypes, Epilepsy, Immunology, Risk factors

## Abstract

Perampanel (PER) is a new-generation antiepileptic drug that has an occasional but significant shortcoming, psychiatric adverse effects (PAEs). Recently, antiepileptic drug-related adverse reactions, such as skin rash and even PAEs, have been discovered to be correlated with certain human leukocyte antigen (HLA) types. Thus, we aimed to analyze specific HLA alleles as risk factors for PER-PAEs. We prospectively enrolled 17 patients with epilepsy who were prescribed PER between May 2016 and Jul 2018 at Seoul National University Hospital and developed PAEs while taking PER. Their HLA types were analyzed compared to those of 19 patients in the PAE-tolerant group and the general Korean population. In silico docking was performed with two different computational programs, AutoDock Vina and SwissDock, to theoretically evaluate the binding affinity of PER in the grooves of the specific HLA alleles. The HLA-DQB1*06:01, DRB1*08:03, and B*54:01 alleles were significantly associated with the patients who developed PER-PAEs compared with the general Korean population (odds ratio [OR] 3.94, p = 0.008, OR 9.24, p = 0.037, and OR 3.25, p = 0.041, respectively). As a haplotype, the combination of the three alleles was significantly more frequent in the PER-PAE group than in both the PER-tolerant group and the general Korean population. DQB1*06:01 and B*54:01 also demonstrated higher docking scores with PER than other alleles. This is the first study to analyze the association of PER-PAEs with specific HLA genotypes. Our results suggest that an HLA-associated genetic predisposition and a possible immunological mechanism are involved in the occurrence of PER-PAEs.

## Introduction

Perampanel (PER) is a new-generation antiepileptic drug (AED) that blocks α-amino-3-hydroxy-5-methyl-4-isoxazolepropionic acid (AMPA)-type glutamate receptors^1–3^. PER has many advantages as a new-generation AED: it has efficacy in drug-resistant focal seizures, a fast loading capacity, few drug-drug interactions, a long half-life, and no critical cutaneous adverse effect as seen with aromatic AEDs^4,5^. Therefore, PER has prevalently been prescribed as a good option in clinical settings.

However, PER has a significant side effect, psychiatric adverse effects (PAEs)^6,7^. In 5–10% of the patients prescribed PER, aggression and irritability have been reported to occur^5,8^. The onset of the symptoms is obscure, but if the symptoms develop, they are hazardous to both the patients and their caregivers; therefore, PER should be discontinued in these patients. However, the pathomechanism of PAEs is largely unknown, making it difficult to prevent symptoms. Given that levetiracetam (LEV)-associated PAEs are associated with specific human leukocyte antigen (HLAs)^9^, we hypothesized that PER-PAEs might have a similar HLA-associated mechanism.

Here, we investigated HLA associations in patients with PER-PAEs. The HLA genotypes of the PER-PAE group were compared to those of the general population as well as the PER-tolerant group. In addition, through in silico analysis, possible immunological reactions were investigated as a pathomechanism of PER-PAEs.

## Method

### Patient enrollment

This study was approved by the Institutional Review Board of the Seoul National University (IRB No 1604-067-754). Written informed consent was obtained from all patients. All methods were carried out in accordance with relevant guidelines and regulations.

Adult patients (age 18–85) who were diagnosed with epilepsy at Seoul National University Hospital were prospectively recruited between May 2016 and Jul 2018 for enrolment in the PER-PAE group. The psychiatric manifestations were evaluated by epilepsy specialists according to the modified version of the Psychiatric Symptoms and Behavior Checklist of the Vanderbilt-Kennedy Center^10^. Psychiatric symptoms included agitation, irritability, aggression, self-injurious behavior, labile mood, impulsivity, and psychosis (delusion, depersonalization, etc.). If psychiatric manifestations were detected, PER was immediately discontinued. PER-PAEs were defined if the patients developed psychiatric symptoms after the last dose titration of PER within 6 weeks, if PER was the only offending drug the patients were exposed to, or if the symptoms disappeared after the discontinuation of PER. Patients with the following conditions were excluded from the study: (1) previous mental illness history, (2) severe mental retardation, (3) severe illness resulting in an inability to evaluate psychiatric symptoms, (4) incomplete clinical data, and (5) an uncertainty of PER as the causative drug. As a control group, the PER-tolerant group and a general Korean population group were compared to the PER-PAE group. The PER-tolerant group was composed of 19 patients who did not show any psychiatric symptoms with a 10 mg dose of PER for 6 months. We used the data of 485 individuals for the general Korean population group^11^.

### HLA genotyping

For DNA extraction, peripheral blood was collected from every patient in both the PER-PAE and PER-tolerant groups. Then, the genotyping of HLA class I and class II genes, including HLA-A, HLA-B, HLA-C, HLA-DRB1, and HLA-DQB1, was performed at the four-digit allele level by direct sequence analysis with established protocols (Biowithus, Seoul, Korea). The previously documented allele frequencies in the Korean population were used as the control group values of the general Korean population.

### In silico docking

In silico docking was performed as previously described^12^. We obtained the three-dimensional structures of PER from the Human Metabolome Database (https://www.hmdb.ca). All HLA subtypes observed in the present study were analyzed in association with PER molecules. The crystallographic structures of HLA-A*02:07, A*11:01, DRB1*11:01, A*24:02, and B*15:01 have been previously determined (Protein Data Bank [PDB] code 3OXS, 4MJ5, 6CPN, 3WL9, 5TXS, respectively). The heterodimeric structures including HLA-DRB1*08:03, DQB1*06:01, B*54:01, C*14:02, C*15:02, DQB1*05:03, DQB1*04:01, and DRB1*04:05 were created using homology modeling with Swiss-Model and UCSF Chimera from the template structures (PDB code 4IS6 for HLA-DRB1, 6DIG for HLA-DQB1, 5TXS for HLA-B, 5VGD for HLA-C) because their crystallographic structures have not been determined experimentally. We used a computational program, AutoDock Vina, and a web-based docking software program, SwissDock (https://www.swissdock.ch/), to calculate the docking score (ΔG) of PER^13,14^.

### Statistics

The difference in the HLA genotype frequencies between the PER-PAE group and the control groups was analyzed with Fisher’s exact test or the Mann–Whitney U test. Haplotypes of the control groups were estimated using Arlequin software (version 3.5.2.2) as previously described^15,16^. Statistical analysis was conducted in R version 3.5.3 (R Foundation for Statistical Computing, Vienna, Austria), and a *p*-value < 0.05 was considered statistically significant.

### Ethical publication statement

We confirm that we have read the Journal’s position on issues involved in ethical publication and affirm that this report is consistent with those guidelines.

## Result

### Characteristics and psychiatric manifestations of patients with PER-PAEs

A total of seventeen patients developed PER-PAEs (Table 1). Of the patients with PER-PAEs, seven (41%) were male, and the median age was 33 years [interquartile range 28–43 years]. Temporal lobe epilepsy was the most common indication for the administration of PER (13, 76%). The patients had suffered epilepsy for 17.6 ± 8.2 years, and until the onset of PAEs, PER was administered for 2.6 ± 2.3 months with a median dose of 4 mg [interquartile range 4–6 mg].

**Table 1:** Demographics and clinical characteristics of the patients with perampanel-induced psychiatric adverse events.

No^a^	Sex	Age	Seizure type	Clinical diagnosis	Baseline seizure frequency	Epilepsy duration (yr)	Concomitant AEDs (mg/d)	Latency (months)	PER dose (mg/day)	Manifestations of PER-PAEs
1	F	28	Focal aware	PLE	1/m	21	CBZ 1,100 dVPA 2,000 ZNS 300 PGB 300 CZP 1.5	1	6	Self-injurious behavior, aggression, impulsivity, psychosis (paranoid delusion)
2	F	32	Focal impaired awareness	TLE	1/m	12	LEV 1,500 ZNS 300 CLB 30	7	8	Self-injurious behavior, impulsivity
3	M	27	Focal impaired awareness	TLE	2/d	27	OXC 1,500 PGB 600 CZP 1.5	3	6	Self-injurious behavior, labile mood, impulsivity
4	F	33	Focal impaired awareness or focal to bilateral tonic–clonic	TLE	8/m	20	CBZ 1,200 LTG 300 ZNS 400	3	4	Psychosis (undescribed delusion)
5	F	36	Focal impaired awareness or focal to bilateral tonic–clonic	TLE	4/m	17	LTG 200 OXC 750 LEV 1,000 VPA 600 PB 90 CLB 10	2	6	Psychosis (depersonalization)
6	F	58	Focal to bilateral tonic–clonic	TLE	3/m	17	OXC 1,800 ZNS 200 PRM 1,000 CLB 20	8	2	Psychosis (persecutory delusion), irritability
7	M	28	Generalized	IGE	1/yr	22	CBZ 800	1	4	Impulsivity
8	F	44	Focal impaired awareness	TLE	2/m	12	ZNS 600	2	6	Aggression, irritability
9	F	34	Focal impaired awareness or focal to bilateral tonic–clonic	TLE	12/m	12	LTG 300 OXC 900 dVPA 1,600 TPM 250 PGB 150 CLB 12.5	4	8	Aggression, irritability
10	F	19	Focal impaired awareness	TLE	3/m	2	LEV 500	3	4	Aggression
11	F	40	Focal impaired awareness	TLE	4/m	16	CBZ 1,000 dVPA 250	1	4	Aggression, irritability
12	M	25	Focal impaired awareness	TLE	3/m	24	OXC 1,200 LEV 1,000 CLB 20	< 1	2	Aggression, agitation, labile mood
13	M	47	Focal impaired awareness	TLE	4/m	22	OXC 1,800 LEV 2,000 PB 180 CLB 10	1	6	Aggression, agitation, loss of interest
14	M	65	Focal to bilateral tonic–clonic	TLE	6/yr	3	RFM 200	< 1	4	Aggression, labile mood
15	M	29	Focal impaired awareness or focal to bilateral tonic–clonic	FLE	10/m	18	VPA 1,500 LEV 1,000	5	8	Aggression
16	F	25	Focal impaired awareness	TLE	3/d	18	PHT 200 LEV 2,000 TPM 400 CLB 10	3	4	Aggression
17	M	43	Focal to bilateral tonic–clonic	FLE	4/yr	36	OXC 1,200 dVPA 1,750	1	4	Aggression

*No* number, *AED* antiepileptic drug, *PER* perampanel, *PER-PAE* perampanel-induced psychiatric adverse event, *F* female, *M* male, *PLE* parietal lobe epilepsy, *TLE* temporal lobe epilepsy, *FLE* frontal lobe epilepsy, *IGE* idiopathic generalized epilepsy, *m* month, *d* day, *yr* year, *CBZ* carbamazepine, *dVPA* divalproex, *ZNS* zonisamide, *PGB* pregabalin, *CZP* clonazepam, *LEV* levetiracetam, *CLB* clobazam, *OXC* oxcarbazepine, *LTG* lamotrigine, *VPA* valproic acid, *PB* phenobarbital, *PRM* primidone, *TPM* topiramate, *RFM* rufinamide, *PHT* phenytoin.

^a^Patients were listed according to representative types of PER-PAEs and significant alleles.

The most common manifestation of PER-PAEs was aggression (11, 65%) (Table 1). For the second most common manifestation of PAEs, irritability, impulsivity, and psychosis were similarly common (4, 24%, respectively). Specific psychoses were as follows: paranoid delusion (Patient 1), depersonalization (Patient 5), persecutory delusion (Patient 6), and undescribed delusion in the medical record (Patient 4). Three patients (18%, Patient 1, 2, 3) attempted self-injurious behavior, and for one patient (Patient 3), it was serious enough to be admitted to the intensive care unit. Additionally, three patients (18%, Patient 3, 12, 14) showed labile mood. Otherwise, two patients (Patient 12, 13) had agitation, and one patient (Patient 13) suffered a loss of interest.

### HLA genotypes and their association with the phenotype of PER-PAE

HLA genotypes were analyzed in seventeen patients with PER-PAEs and in nineteen patients with PER tolerance. Detailed four-digit HLA alleles of the patients with PER-PAEs are described in Table 2 and Supplementary Table S1.

**Table 2:** Human leukocyte antigen genotypes of patients with perampanel-induced psychiatric adverse events.

No^a^	Representative PER-PAE	HLA-A	HLA-B	HLA-C	HLA-DQB1	HLA-DRB1
1	Self-injurious behavior	30:01/30:01	13:02/**54:01**	01:02/06:02	**06:01**/06:03	**08:03**/13:01
2		02:07/24:02	40:02/46:01	01:02/03:04	03:01/**06:01**	**08:03**/11:01
3		11:01/33:03	15:01/58:01	03:02/04:01	03:01/03:02	04:01/04:06
4	Psychosis	24:02/26:01	**54:01**/55:02	01:02/01:02	03:02/**06:01**	08:02/**08:03**
5		02:07/24:02	39:01/46:01	01:02/07:02	**06:01**/**06:01**	**08:03**/**08:03**
6		32:01/33:03	44:02/58:01	03:02/05:01	02:01/03:01	03:01/12:01
7	Impulsivity	11:01/24:02	51:01/52:01	12:02/14:02	03:03/**06:01**	09:01/15:02
8	Aggression	02:01/33:03	15:18/**54:01**	07:04/14:03	04:01/**06:01**	04:05/**08:03**
9		02:06/11:01	51:01/52:01	12:02/14:02	04:02/**06:01**	04:10/15:02
10		24:02/24:02	07:02/**54:01**	01:02/07:02	05:01/**06:01**	01:01/**08:03**
11		02:03/02:06	07:02/38:02	07:02/07:02	05:01/05:01	01:01/15:02
12		11:01/11:02	**54:01**/55:02	01:02/12:03	03:01/05:03	11:01/14:05
13		31:01/33:03	40:06/58:01	03:02/08:01	03:01/06:09	12:02/13:02
14		02:01/26:01	15:11/51:01	03:03/03:04	03:01/05:02	11:01/14:54
15		02:01/02:06	15:38/40:01	03:03/03:04	03:02/06:02	04:06/15:01
16		02:07/11:01	51:01/51:01	14:02/14:02	03:01/06:02	11:01/15:01
17		30:04/31:01	14:01/51:01	08:02/14:02	04:02/06:02	04:04/15:01

*No* number, *PER-PAE* perampanel-induced psychiatric adverse event, *HLA* human leukocyte antigen.

Bold typefaces indicate alleles that increased significantly in this PER-PAE group. See Table 3.

^a^Patients were listed according to representative types of PER-PAEs and significant alleles.

Among the alleles, the frequencies of DQB1*06:01 and B*54:01 were significantly higher in the PER-PAE group than in the general Korean population (p = 0.008, odds ratio [OR] 3.94, 95% confidence interval [CI] 1.47–11.60, p = 0.041, OR 3.25, 95% CI 1.06–9.52, respectively) but not in the PER-tolerant group (Table 3). In addition, the HLA-DRB1*08:03 allele also showed a significantly higher genotype frequency in the PER-PAE group than in both the PER-tolerant group (p = 0.037, OR 9.24, 95% CI 1.14–234.18) and the general Korean population (p = 0.041, OR 2.97, 95% CI 1.06–8.34).

**Table 3:** Association between four-digit HLA alleles and perampanel-induced psychiatric adverse events.

HLA allele or haplotype	Phenotype frequency			PER-PAE versus PER-tolerant		PER-PAE versus general population		PER-tolerant versus general population		Docking scores in –ΔG	
	PER-PAE (% of 17)	PER-tolerant (% of 19)	General population (% of 485)	OR (95% CI)	P value	OR (95% CI)	P value	OR (95% CI)	P value	AutoDock Vina	SwissDock
**Prevalent alleles or haplotypes in the PER-PAE group**											
DQB1*06:01	8 (47%)	3 (16%)	89 (18%)	4.53 (0.90–24.61)	0.070	3.94 (1.47–11.60)	0.008^a^	0.83 (0.20–2.88)	> 0.999	10.7	8.0
DRB1*08:03	6 (35%)	1 (5%)	75 (15%)	9.24 (1.14–234.18)	0.037^a^	2.97 (1.06–8.34)	0.041^a^	0.30 (0.01–2.01)	0.333	8.2	7.6
B*54:01	5 (29%)	3 (16%)	55 (11%)	2.17 (0.42–12.39)	0.434	3.25 (1.06–9.52)	0.041^a^	1.46 (0.35–4.91)	0.471	9.6	7.8
A*02:07	3 (18%)	1 (5%)	29 (6%)	3.72 (0.37–104.92)	0.326	3.36 (0.78–12.62)	0.087	0.87 (0.04–5.43)	> 0.999	8.0	7.6
A*11:01	5 (29%)	1 (5%)	100 (21%)	7.11 (0.75–183.93)	0.081	1.60 (0.53–4.55)	0.369	0.21 (0.01–1.40)	0.143	9.0	7.9
DRB1*11:01	4 (24%)	2 (11%)	43 (9%)	2.55 (0.41–21.63)	0.391	3.15 (0.91–10.05)	0.065	1.21 (0.19–5.16)	0.683	8.1	7.6
Haplotype #1^b^	6 (35%)	1 (5%)	66 (14%)	9.24 (1.14–234.18)	0.037^a^	3.45 (1.22–9.74)	0.024^a^	0.35 (0.02–2.35)	0.492		
Haplotype #2^b^	4 (24%)	0	10 (2%)	Inf (1.11–Inf)	0.040^a^	14.37 (3.73–56.51)	0.001^a^	0 (0–11.96)	> 0.999		
**Prevalent alleles in the PER-tolerant group**											
C*14:02	4 (24%)	6 (32%)	61 (13%)	0.67 (0.15–3.12)	0.717	2.13 (0.62–6.62)	0.257	3.20 (1.16–9.35)	0.029^a^	8.8	7.2
C*15:02	0	3 (16%)	25 (5%)	0 (0–1.84)	0.231	0 (0–4.30)	> 0.999	3.44 (0.80–12.60)	0.082	7.3	7.0
A*24:02	5 (29%)	11 (58%)	188 (39%)	0.31 (0.07–1.27)	0.106	0.66 (0.22–1.87)	0.613	2.17 (0.84–5.59)	0.101	9.2	7.0
DQB1*05:03	1 (6%)	4 (21%)	46 (9%)	0.24 (0.01–2.12)	0.342	0.60 (0.03–3.65)	> 0.999	2.54 (0.75–7.93)	0.109	7.9	7.8
DQB1*04:01	1 (6%)	5 (26%)	80 (16%)	0.18 (0.01–1.74)	0.182	0.32 (0.02–1.95)	0.332	1.81 (0.61–5.40)	0.343	9.5	7.8
DRB1*04:05	1 (6%)	5 (26%)	83 (17%)	0.18 (0.01–1.74)	0.182	0.30 (0.01–1.87)	0.329	1.73 (0.58–5.15)	0.350	8.1	7.6
B*15:01	1 (6%)	4 (21%)	93 (19%)	0.24 (0.01–2.12)	0.342	0.26 (0.01–1.79)	0.218	1.12 (0.34–3.59)	0.771	9.2	7.1

*HLA* human leukocyte antigen, *PER-PAE* perampanel-induced psychiatric adverse event, *PER* perampanel, *OR* odds ratio, *CI* confidence interval.

^a^P value < 0.05.

^b^Haplotype #1, HLA-DQB1*06:01-DRB1*08:03, Haplotype #2: HLA-B*54:01-DQB1*06:01-DRB1*08:03 or HLA-B*54:01-DRB1*08:03.

As a haplotype, concomitant DQB1*06:01 and DRB1*08:03 alleles showed significantly higher frequency in the PER-PAE group than in both the PER-tolerant group (p = 0.037, OR 9.24, 95% CI 1.14–234.18) and the general Korean population (p = 0.024, OR 3.45, 95% CI 1.22–9.74) (Table 3). The combination of the alleles DQB1*06:01, DRB1*08:03, and B*54:01 was also significantly more frequent both in the PER-tolerant group (p = 0.040, OR Inf, 95% CI 1.11–Inf) and in the general Korean population (p = 0.014, OR 14.37, 95% CI 3.73–56.51). Remarkably, haplotype HLA-DQB1*06:01-DRB1*08:03 had a tendency to be more frequent in the patients with more severe PER-PAEs; four of 6 patients (66.7%) who showed self-injurious behavior or psychosis concomitantly had the DQB1*06:01 and DRB1*08:03 alleles (Table 2).

### In silico docking

**Figure 1:**
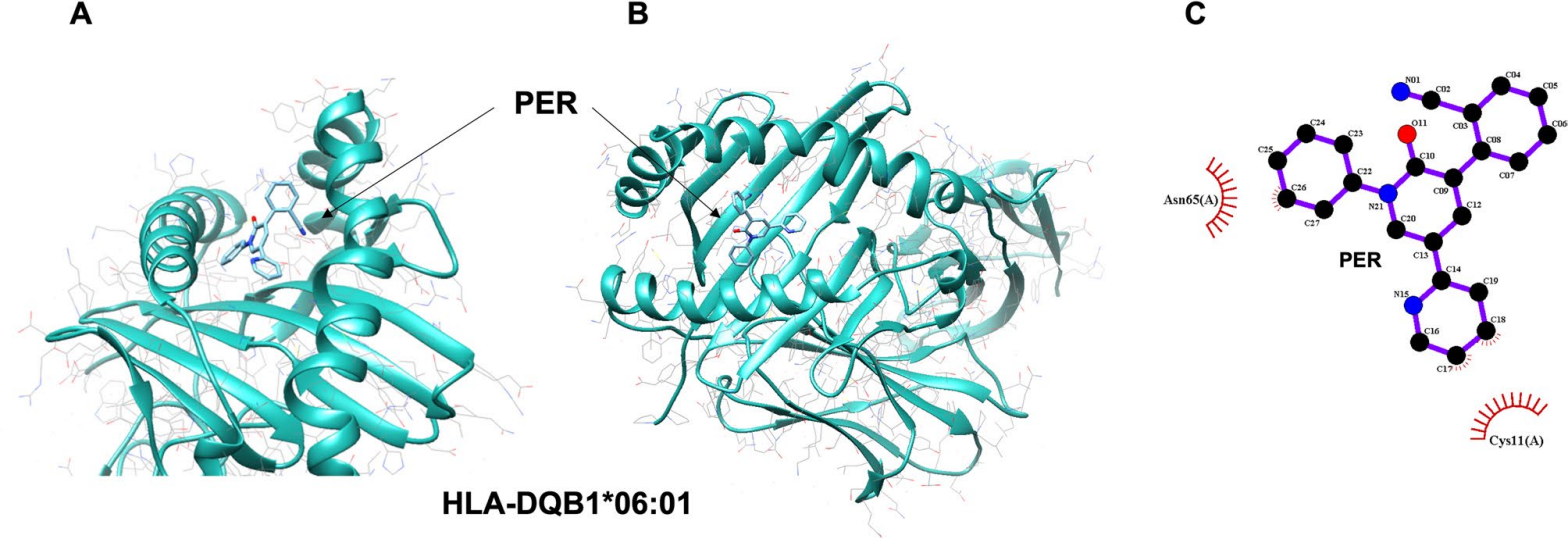
In silico modeling of the molecular interaction between HLA-DQB1*06:01 and PER. (**A**,**B**) The PER molecule was predicted to be docked into the middle of the groove of HLA-DQB1*06:01 with higher affinity (− 10.7 kcal/mol). (**C**) Specific plot of the interaction between ligand (PER) and receptor (HLA-DQB1*06:01) using LigPlot + v2.1. *HLA* human leukocyte antigen, *PER* perampanel.

The binding affinity of the PER molecule to HLA alleles calculated by two different software programs is displayed in Table 3. HLA alleles causing PER-PAEs tended to have higher docking scores than alleles prevalent in the PER-tolerant group. In detail, PER was predicted to be docked into the groove of DQB1*06:01 (Fig. 1) with a docking score of 10.7, which was higher than the docking scores of DQB1*05:03 and DQB1*04:01 (docking scores (− kcal/mol) 7.9 and 9.5, respectively, in AutoDock Vina). B*54:01 had a higher binding affinity than B*15:01 (docking scores (− kcal/mol) 9.6 vs. 9.2 in AutoDock Vina and 7.8 vs. 7.1 in SwissDock).

However, DRB1*08:03, the allele significantly more frequent in the PER-PAE group than in both the PER-tolerant group and the general Korean population, did not show stronger binding than DRB1*04:05 (docking scores (kcal/mol) 8.2 vs. 8.1 in AutoDock Vina and 7.6 vs. 7.6 in SwissDock).

## Discussion

This is the first study to analyze the association of PER-PAEs with specific HLA genotypes. We demonstrated that the HLA-DQB1*06:01, DRB1*08:03, and B*54:01 alleles were associated with PER-PAEs. As a haplotype, the combination of the three alleles was significantly more frequent in the PER-PAE group than in both the PER-tolerant group and the general Korean population. Among them, DQB1*06:01 might be the allele most susceptible to PER-PAEs, since it was more frequent in the patients with more severe PAEs and had a higher docking score with PER than other alleles. Our research implies that HLA-associated genetic susceptibility could be involved in the occurrence of PER-PAEs.

In our study, the PER-PAEs were categorized according to the modified version of the Psychiatric Symptoms and Behavior Checklist of the Vanderbilt-Kennedy Center. Aggression was the most common PAE observed in our patients taking PER. This is consistent with previous reports^6,7^, and aggression dramatically increases the burden of epilepsy on patients and caregivers. Impulsivity, one of the second most common symptoms, becomes extremely dangerous when it is accompanied by self-injurious behavior^17,18^. Indeed, all three patients who attempted self-injurious behavior simultaneously showed impulsivity, and one male patient even attempted suicide, which resulted in his admittance to an intensive care unit. Therefore, in regard to the patients’ safety and caregivers’ burden, the importance of researching the risk factors for PER-PAEs cannot be emphasized enough.

Specific HLA genotypes are known to be risk factors for several AED adverse reactions. For instance, we have demonstrated the association between the cutaneous adverse reaction induced by lamotrigine and oxcarbazepine with certain HLA types^12,19^. By showing in silico docking of the AED in the HLA groove, we have suggested the possibility of oxcarbazepine acting as a hapten in the allergic reaction. In the same regard, it is remarkable that PER-PAEs had significant associations with the HLA-DQB1*06:01, DRB1*08:03, and B*54:01 alleles, as LEV-PAEs were correlated with the specific HLA genotype A*11:01^9^. This implies that PAEs that develop in the brain could also be idiosyncratic adverse reactions induced by an immunological response. In patients with autoimmune encephalitis such as *N*-methyl-d-aspartate receptor-encephalitis and leucine-rich glioma-inactivated protein1-antibody encephalitis, it has been demonstrated that psychiatric manifestations can be induced by an autoimmune mechanism in the brain^10,20,21^. It might not be a coincidence that an association of house-dust-mite-sensitive allergic rhinitis and the same HLA genotypes, DQB1*06:01 and DRB1*08:03, was demonstrated in Chinese subjects^22^. Moreover, in the Korean population, B*54:01 was associated with Kawasaki disease, an acute systemic vasculitis causing heart disease in children^23^.

The in silico docking analysis supports this hypothesis. Remarkably, the docking scores of DRB1*06:01 and B*54:01 were higher than those of the other alleles that were prevalent in the PER-tolerant group. Since the docking score indirectly indicates a binding affinity of an antigen to HLA grooves, the higher scores imply stronger binding of PER to the particular HLA as the antigen or hapten causing the immunological reaction. It is notable that two different programs, AutoDock Vina and SwissDock, showed similar results for the docking scores. Thus, our analysis would reflect more objective and realistic results. On the other hand, the score of DRB1*08:03 was not much higher than those of the other alleles. DRB1*08:03 might be just a bystander of DQB1*06:01 or B*54:01, not the main cause of the immunological reaction that induces PER-PAEs. Indeed, both alleles, DQB1*06:01 and B*54:01, have high phenotypic frequencies (14% for HLA-DQB1*06:01-DRB1*08:03 and 2% for HLA-B*54:01-DQB1*06:01-DRB1*08:03, respectively) when composing the haplotype with DRB1*08:03 in the Korean population^11^. In our study, the haplotype analysis further demonstrated that HLA-DQB1*06:01-DRB1*08:03 and HLA-B*54:01-DQB1*06:01-DRB1*08:03 were concomitantly associated with PER-PAEs.

According to the high docking scores, the alleles DQB1*06:01 or B*54:01 could be the main risk factors involved in the PAE pathomechanism. However, it is remarkable that the two alleles have different antigen processing; DQB1*06:01 is a major histocompatibility complex (MHC) class II allele, interacting with CD4^+^ T cells through extracellular antigen processing, and B*54:01 is an MHC class I allele, interacting with CD8^+^ T cells through intracellular processing. In the clinic, CD4^+^ T cell involvement might be more plausible, in that PER-PAEs are fully reversible if the AED is discontinued, than a cytotoxic CD8^+^ T cell response, which results in irreversible damage to the target. Actually, for AED-related cutaneous adverse drug reactions, MHC class II molecules are associated with relatively mild maculopapular eruption, but MHC class I molecules are significantly correlated with an irreversible skin rash such as Stevens–Johnson syndrome and toxic epidermal necrolysis^12,24^. Although DQB1*06:01 was associated with more severe clinical manifestations, it would be noteworthy that the patients with PER-PAEs fully recovered without any sequelae after the discontinuation of PER.

Taken together, among the three alleles, we cautiously suggest that DQB1*06:01 could be the allele most susceptible to PER-PAEs. In addition to its high docking score and high frequency in the patients with PER-PAEs, DQB1*06:01 was more common in the patients with more severe psychiatric manifestations, such as self-injurious behavior or psychosis, and the pathomechanism of MHC class II molecules might be more plausible than that of MHC class I molecules in that the clinical manifestation was fully reversible. Nevertheless, we should not completely exclude the possibility that HLA-B*54:01 and DRB1*08:03 are involved in the pathomechanism of PER-PAEs; DRB1*08:03 had a higher odds ratio in the PER-PAE group than in the PAE-tolerant group. Moreover, as previous results have shown, LEV-PAEs were associated with MHC class I molecules^9^, and it is not yet known exactly how AED-related cerebral adverse effects occur at the cellular level. The immune reaction in the central nervous system would be much more complex than in the skin. Beside the antigen presentation and CD8^+^ T cell activation, MHC class I molecules are known to perform nonclassical functions in association with multiple cytokines and may be engaged in controlling neuronal plasticity^25–29^. We hypothesize that the nonclassical function of MHC class I molecule could also be associated with a reversible phenotype, but this should be investigated in further studies. Future analysis with genome-wide association studies might also play a critical role in the identification of which HLA alleles would be important for PER-PAEs.

The study is limited by the small number of patients who were included in the PER-PAE group and the PAE-tolerant group. In addition, the analysis was not controlled for co-medications. However, to overcome this limitation, patients with PER-PAEs were strictly selected according to certain criteria, and the PER-tolerant group was qualified to be an extreme phenotype with a daily dose of 10 mg per day for 6 months. Moreover, the PER-PAEs were analyzed using an objective tool, the modified version of the Psychiatric Symptoms and Behavior Checklist of the Vanderbilt-Kennedy Center. The results will need to be validated in an independent large cohort. Lastly, the HLA allele frequency differs according to ethnicity so that further investigation is required in multiple ethnic groups.

In summary, our study shows that PER-PAEs were associated with HLA-DQB1*06:01, DRB1*08:03, and B*54:01 and their combinations as haplotypes in the Korean population. Among the alleles, DQB1*06:01 could be the most likely HLA type correlated with the immunological reaction of PER-PAEs. As HLA-related genetic risk factors for each ethnicity are important in and of themselves, the results of this study would be helpful as a reference to investigate potential screening methods for Korean patients who will be prescribed PER. Moreover, our results of the HLA-related genetic susceptibility of PER-PAEs suggest that psychiatric symptoms induced by adverse drug effects could be induced by an immunological reaction in the brain. Therefore, further research should be performed with a larger number of patients in terms of the pathomechanism of PAEs in the near future.

## Supplementary information

Supplementary Information

## Data Availability

The data that support the findings of this study are available from the corresponding author upon reasonable request.
