# Supplementary material for: HLAs associated with perampanel-induced psychiatric adverse effects in a Korean population
Source: Sci Rep. 2020 Aug 12;10:13667. doi: 10.1038/s41598-020-70601-1 (PMC7423598; doi:10.1038/s41598-020-70601-1)
Supplement: Supplementary file 1 — Supplementary Information [file 41598_2020_70601_MOESM1_ESM.docx]

**HLAs associated with perampanel-induced psychiatric adverse effects in a Korean population**

Yoonhyuk Jang, MD^1†^, Tae-Joon Kim, MD^2†^, Jangsup Moon, MD, PhD^1,3, †^, Tae-Won Yang, MD^4^, Keun Tae Kim, MD^5^, Byeong-Su Park, MD^6^, Jung-Ah Lim, MD^7^, Jin-Sun Jun, MD^8^, Soon-Tae Lee, MD, PhD^1^, Keun-Hwa Jung, MD, PhD^1^, Kyung-Il Park, MD, PhD^1,9^, Ki-Young Jung, MD, PhD^1^, Kon Chu, MD, PhD^1*^, Sang Kun Lee, MD, PhD^1*^

*^1^Department of Neurology, Laboratory for Neurotherapeutics, Comprehensive Epilepsy Center, Biomedical Research Institute, Seoul National University Hospital, Seoul, South Korea*

*^2^Department of Neurology, Ajou University School of Medicine, Suwon, South Korea*

*^3^Rare Disease Center, Seoul National University Hospital, Seoul, South Korea*

*^4^Department of Neurology, Gyeongsang National University Changwon Hospital, Gyeongsang National University School of Medicine, Changwon, Republic of Korea*

*^5^Department of Neurology, Keimyung University Dongsan Medical Center, Daegu, Republic of Korea*

*^6^Department of Neurology, Ulsan University Hospital, Ulsan, Republic of Korea*

*^7^Department of Neurology, Chamjoeun Hospital, Gwangju, Republic of Korea*

*^8^Department of Neurology, Kangnam Sacred Heart Hospital, Hallym University College of Medicine, Seoul, South Korea*

*^9^Department of Neurology, Seoul National University Hospital Healthcare System Gangnam Center, Seoul, South Korea*

^†^ These authors contributed equally to this study as co-first authors.

^*^ These authors contributed equally to this study as co-corresponding authors.

*Character count for the title: 90*

*Total word count for the abstract: 244*

*Total word count for the manuscript:2498*

*Number of references: 24*

*Number of tables: 3*

*Number of figures: 1*

**Running head: specific HLAs as risk factors for Perampanel induced Psychiatric Adverse Effects**

**ORCID IDs**

Yoonhyuk Jang: 0000-0002-3346-3357

Tae-Joon Kim: 0000-0001-8451-6634

Jangsup Moon: 0000-0003-1282-4528

Kon Chu: 0000-0001-5863-0302

Sang Kun Lee: 0000-0003-1908-0699

**Correspondence:**

Kon Chu, MD, PhD

Department of Neurology, Seoul National University Hospital,

101 Daehak-ro, Jongno-gu, Seoul 110-744, South Korea

Tel.: +82-2-2072-1878/Fax: + 82-2-3672-7553

Email: [stemcell.snu@gmail.com](mailto:stemcell.snu@gmail.com)

and

Sang Kun Lee, MD, PhD

Department of Neurology, Seoul National University Hospital,

101 Daehak-ro, Jongno-gu, Seoul 110-744, Korea

Tel: +82-2-2072-2923/Fax: +82-2-3672-7553

E-mail: [sangkun2923@gmail.com](mailto:sangkun2923@gmail.com)

Supplementary Table 1. Human leukocyte antigen genotypes of the patients in perampanel-tolerant group

| No | HLA-A | HLA-B | HLA-C | HLA-DQB1 | HLA-DRB1 |
| --- | --- | --- | --- | --- | --- |
| 1 | 24:02/24:02 | 07:02/07:02 | 07:02/07:02 | 04:01/05:01 | 01:01/04:05 |
| 2 | 02:01/33:03 | 15:01/58:01 | 03:02/08:01 | 02:01/06:02 | 03:01/15:01 |
| 3 | 24:02/33:03 | 07:02/40:01 | 03:04/07:02 | 05:01/05:03 | 01:01/14:54 |
| 4 | 02:01/31:01 | 27:05/54:01 | 01:02/01:02 | 05:01/05:03 | 01:01/14:05 |
| 5 | 24:02/24:02 | 07:02/35:01 | 03:04/07:02 | 05:01/06:02 | 01:01/15:01 |
| 6 | 24:02/26:01 | 51:01/54:01 | 01:02/14:02 | 03:03/05:03 | 12:01/14:05 |
| 7 | 02:01/31:01 | 13:01/54:01 | 01:02/03:04 | 03:01/04:01 | 04:05/12:02 |
| 8 | 03:01/24:02 | 44:02/51:01 | 05:01/14:02 | 04:01/04:02 | 04:05/08:02 |
| 9 | 24:02/24:02 | 13:01/51:01 | 03:04/14:02 | 03:03/06:01 | 08:03/09:01 |
| 10 | 02:01/31:01 | 51:02/67:01 | 07:02/15:02 | 03:01/06:02 | 12:01/15:01 |
| 11 | 02:01/24:02 | 48:01/52:01 | 08:01/12:02 | 03:01/06:01 | 11:01/15:02 |
| 12 | 24:02/33:03 | 44:03/52:01 | 07:01/12:02 | 02:02/06:01 | 07:01/15:02 |
| 13 | 24:02/30:01 | 13:02/15:01 | 06:02/15:02 | 02:02/03:01 | 07:01/11:01 |
| 14 | 02:06/11:01 | 15:01/58:01 | 03:02/04:01 | 02:01/03:02 | 03:01/04:06 |
| 15 | 02:01/31:01 | 40:01/51:01 | 14:02/15:02 | 04:01/04:01 | 04:05/04:05 |
| 16 | 02:06/24:02 | 35:01/59:01 | 01:02/08:03 | 03:01/04:01 | 04:05/14:03 |
| 17 | 02:01/26:01 | 15:01/51:01 | 03:03/14:02 | 03:01/06:04 | 12:02/13:02 |
| 18 | 24:02/30:04 | 14:01/35:01 | 04:01/08:02 | 04:02/05:03 | 04:04/0.620 |
| 19 | 02:06/02:07 | 46:01/51:01 | 01:02/14:02 | 03:01/03:02 | 12:01/12:02 |

No, number; HLA, human leukocyte antigen
